# Therapeutic and Preventive Effects of Osteoclastogenesis Inhibitory Factor on Osteolysis, Proliferation of Mammary Tumor Cell and Induction of Cancer Stem Cells in the Bone Microenvironment

**DOI:** 10.3390/ijms19030888

**Published:** 2018-03-16

**Authors:** Mitsuru Futakuchi, Takao Nitanda, Saeko Ando, Harutoshi Matsumoto, Eri Yoshimoto, Katsumi Fukamachi, Masumi Suzui

**Affiliations:** 1 Department of Pathology, Nagasaki University Hospital, Nagasaki 851-8501, Japan; 2 Department of Pathology, Nagasaki University Graduate School of Biomedical Sciences, Nagasaki 852-8523, Japan; nitanda@nagasaki-u.ac.jp; 3 Department of Molecular Toxicology, Graduate School of Medical Sciences, Nagoya City University, Nagoya 467-8601, Japan; c161702@ed.nagoya-cu.ac.jp (S.A.); matsu88@med.nagoya-cu.ac.jp (H.M.); eyoshimo@med.nagoya-cu.ac.jp (E.Y.); kfukamac@med.nagoya-cu.ac.jp (K.F.); suzui@med.nagoya-cu.ac.jp (M.S.)

**Keywords:** bone metastasis, mammary tumor, RANKL, bone microenvironment, osteoclastogenesis inhibitory factor, cancer stem cell

## Abstract

Background: We examined the effects of recombinant human osteoclastogenesis inhibitory factor (hOCIF) on osteolysis, proliferation of mammary tumor cells, and induction of cancer stem cells (CSCs) in the tumor-bone and tumor-subcutaneous microenvironments (TB- and TS-microE). Methods: Mouse mammary tumor cells were transplanted onto the calvaria or into a subcutaneous lesion of female mice, creating a TB-microE and a TS-microE, and the mice were then treated with hOCIF. To investigate the preventive effects of hOCIF, mice were treated with hOCIF before tumor cell implantation onto the calvaria (Pre), after (Post), and both before and after (Whole). The number of CSCs and cytokine levels were evaluated by IHC and ELISA assay, respectively. Results: hOCIF suppressed osteolysis, and growth of mammary tumors in the TB-microE, but not in the TS-microE. In the Pre, Post, and Whole groups, hOCIF suppressed osteolysis, and cell proliferation. hOCIF increased mouse osteoprotegrin (mOPG) levels in vivo, which suppressed mammary tumor cell proliferation in vitro. These preventive effects were observed in the dose-dependent. hOCIF did not affect the induction of CSCs in either microenvironment. Conclusion: While receptor activator of NF-κB ligand (RANKL) targeting therapy may not affect the induction of CSCs, RANKL is a potential target for prevention as well as treatment of breast cancer bone metastasis.

## 1. Introduction

Breast cancer is the leading cause of malignancy in Japanese women [1] and the second leading cause of malignancy in American women [2]. Early detection of breast cancer has increased the 5-year survival rate to more than 85% of diagnosed patients, and Japanese patients have significantly better survival than all other races [3]. However, progression to bone reduces the 5-year survival rate to 26% due to the limited curative treatments available [4,5].

RANKL, which signals through receptor activator of NF-κB (RANK) on preosteoclasts was shown to induce differentiation and activation leading to bone resorption [6]. Receptor activator of NF-κB ligand (RANKL) has also been shown to be modified by proteases to favor tumor progression [7,8]. Previously, we demonstrated that matrix metalloproteinase 7 (MMP7) could cleave membrane-bound RANKL into a soluble form (sRANKL), which abate the contact-dependent nature of osteoblast-osteoclast interaction and promote osteoclast activation and subsequent osteolysis in a prostate cancer model [9]. In breast cancer model, we also demonstrated that cathepsin G would shed RANKL, generating sRANKL, which could differentiate and activate osteoclast precursors [10]. Osteolysis mediated by activated osteoclasts releases bone-derived growth factors and promotes the survival and proliferation of tumor cells [6,11]. Osteoclastogenesis inhibitory factor (OCIF) (also known as osteoprotegerin (OPG) and tumor Necrosis Factor (Ligand) Superfamily, Member 11B, TNFSF11B) is a soluble member of the tumor necrosis factor receptor family of proteins and acts as decoy receptor for TNFSF11/RANKL. Thus, OCIF plays an important role in the negative regulation of osteoclastic bone resorption.

Bone metastases of breast cancer is associated with results skeletal-related events, SREs, such as bone pain, pathologic fracture, hypercalcemia, and spinal instability [12,13]. The management of bone metastases and the resulting SREs can be accomplished by the combination of analgesic agents, chemotherapy, radiation therapy and bone modifying agents (BMAs) such as recombinant OCIF, RANKL antibody or bisphosphonate [14]. Clinical trials revealed that these BMAs increased the time to SRE and reduced the SREs of breast cancer patients with bone metastasis [15]. Currently, BMAs are considered as the standard of care for managing SREs in patients with bone metastatic diseases [16,17], while BMAs did not significantly prolong the survival of the overall study population.

Because of the improvements in cancer therapies for solid tumors, patients with metastatic disease are living longer, and for these patients there is a need to preserve the quality of life (QOL) for an extended period of time. Thus, there is increasing focus on strategies in the management of metastatic bone diseases to delay worsening of skeletal pain and aggravation of metastatic bone diseases. Early palliative treatment with bone modifying agents has enhanced the QOL in patients with metastatic lung cancer and resulted in the reduction of aggressive end-of-life care [18,19].

Therapy resistance represents a significant hurdle in the treatment of breast cancer forcing the development of alternative strategies. Recent cancer stem cell (CSC) theory became a trigger to set the hypothesis for tumor development and progression. Accumulating evidence suggests that CSCs closely correlate with tumor metastasis [20,21] and are responsible for tumor proliferation, metastasis, tumor relapse, and resistance to chemo- and radiation therapy [22,23].

To investigate the effects of hOCIF in the tumor microenvironment, we examined the tumor growth, osteoylsis, and induction of CSC using our breast cancer bone invasion model [10,24]. In the present study, we transplanted mouse mammary tumor cells onto the cranial bone and into a subcutaneous lesion in mice, and then treated the mice with recombinant human TNFRSF11B: In this study, we refer to recombinant human TNFRSF11B as hOCIF and endogenouse mouse TNFRSF11B as mouse osteoprotegrin (mOPG). To investigate the mechanism underlying the inhibitory action of hOCIF, we analyzed the levels of transforming growth factor β (TGFβ), RANKL, and mOPG in the tumor microenvironments.

## 2. Results

### 2.1. Effects of hOCIF on Tumor Growth in the Tumor Microenvironments

**Figure 1:**
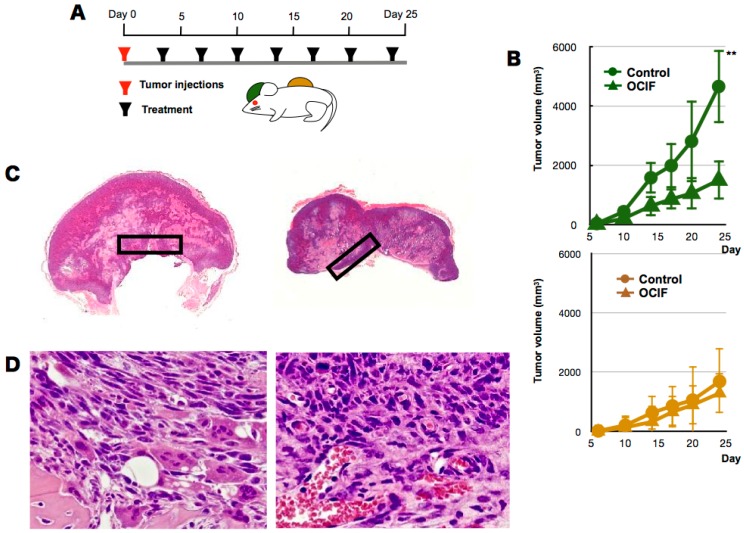
Effects of hOCIF on tumor growth in the tumor microenvironments (1). (**A**) To examine the effects of hOCIF on our mouse mammary tumor-bone invasion model, we implanted mouse mammary tumor cell lines into two different sites, the cranial bone and a subcutaneous lesion, and then injected mice with hOCIF six times over the course of the experimental period; (**B**) We compared the growth of the transplanted tumor on the cranial bone (upper) and subcutaneous lesion (lower) in treated and untreated mice; (**C**) The tumor bone interface (TB-interface, magnification ×1, left) and tumor subcutaneous interface (TS-interface, magnification ×1, right) in the cranial tumor and in the subcutaneous tumor, respectively (black square); (**D**) Histological analysis of the TB-interface revealed strong osteolysis associated with the induction of numerous osteoclasts in the TB-microE (left, ×400). Tumor cells were growing with micro vessel invasion at the TS-microE (right, ×400). ** *p* < 0.01 vs. Con at TB-Interface.

**Figure 2:**
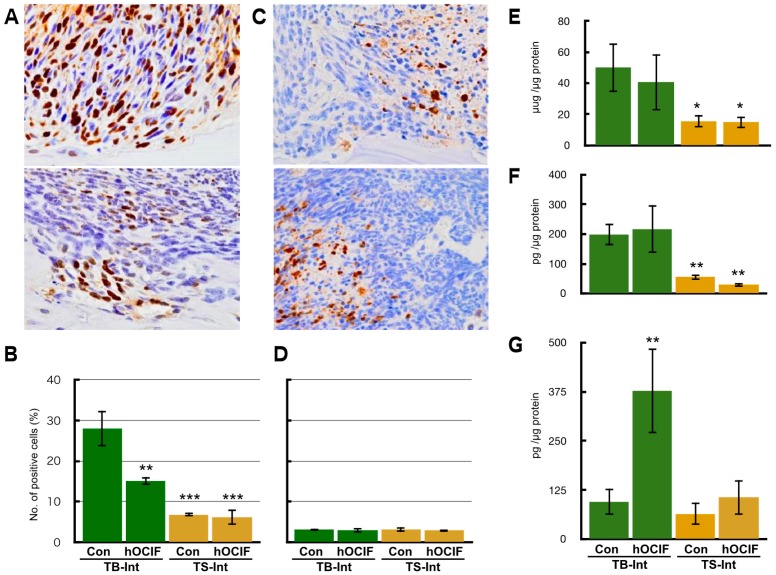
Effects of hOCIF on tumor growth in the tumor microenvironments (2). (**A**) PCNA staining of the control group at the TB-interface (upper, ×400) and the treatment group (lower, ×400); (**B**) Quantitative analysis of PCNA positive cells at the TB- and TS-interfaces; (**C**) Cleaved Caspase 3 staining of the control group at the TB-interface (left, ×400) and TS-interface (right, ×400); (**D**) Quantitative analysis of cleaved caspase 3 positive cells at the TB- and TS-interfaces; Cytokines levels of TGFβ (**E**), RANKL (**F**), and mOPG (**G**) at the TB- and TS-interfaces. The levels of TGFβ and RANKL level were higher at the TB-interface compared with those at the TS-interface. hOCIF treatment did not suppressed the levels of these cytokines. hOCIF treatment significantly increased mOPG levels at the TB-interface but did not change mOPG levels at the TS-interface (**G**). *, **, *** *p* < 0.05, *p* < 0.01, *p* < 0.001 vs. Con at the TB-Interface.

To examine the effects of hOCIF on our mouse mammary tumor-bone invasion model, we implanted mouse mammary tumor cell lines into two different sites in BALB/c mice, the dorsal skin flap over the calvaria and into a subcutaneous lesion, and then injected the mice six times with hOCIF over the course of the experimental period (Figure 1A). We compared the growth of the transplanted tumor on the cranial bone and in the subcutaneous lesion in treated and untreated mice (Figure 1B). A steep increase in the tumor size was observed on the cranial bone, and hOCIF treatment significantly suppressed the tumor growth (Figure 1B upper). In contrast, the tumor grew much more slowly in the subcutaneous lesions, and hOCIF treatment did not suppress tumor growth (Figure 1B lower). In these mice, the tumor-bone interface (TB-interface) (Figure 1C left) and tumor-subcutaneous interface (TS-interface) (Figure 1C right) can be readily delineated (Figure 1C). Strong osteolysis associated with induction of numerous osteoclasts was observed at the TB-interface (Figure 1D left). At the TS-interface, the tumor cells grew with micro vessel invasion (Figure 1D right). We observed tumor cells strongly positive for proliferating cell nuclear antigen (PCNA) at the TB-interface in the control group (Figure 2A upper), and hOCIF treatment significantly reduced the number of PCNA positive cells at the TB-interface (Figure 2A lower, Figure 2B left). Fewer tumor cells strongly positive for PCNA were observed at the TS-interface in the control group and hOCIF treatment did not significantly reduce the number of PCNA positive cells (Figure 2B right). Thus, hOCIF treatment suppressed the tumor growth in the TB-microE but did not suppress that in the TS-microE.

We also examined the induction of tumor cell apoptosis. We observed the tumor cells strongly positive for cleaved caspase 3 at the TB-interface (Figure 2C upper), and TS-interface (Figure 2C lower) in the control group. hOCIF treatment did not reduce the number of cleaved caspase 3 positive cells in the TB- or TS-interfaces (Figure 2D). Thus, hOCIF treatment did not induce apoptosis in the tumors in the TB- or TS-microE.

### 2.2. Effects of hOCIF on Osteolysis and Cytokine Levels in the Tumor Microenvironments

We evaluated the effects of hOCIF on osteolysis, induction of osteoclasts (Supplement Figure S1), and the levels of cytokines that are related to bone metastasis (Figure 2E–G). Since we observed defects of the cranial bone, the severity of bone destruction was examined by the ratio of the length of bone destruction to that of the cranial bone (bone destruction index) (Supplement Figure S1A). Quantitative analysis of the bone destruction index revealed that hOCIF significantly suppressed the degree of osteolysis associated with mammary tumor growth at the TB-interface (Supplement Figure S1B). In agreement with this result, at the TB-interface of the control group, numerous osteoclasts positive for Tartrate-Resistant Acid Phosphatase (TRAP) staining were observed (Supplement Figure S1C), and hOCIF treatment significantly reduced the number of these osteoclasts (Supplement Figure S1D,E).

Next, we measured the levels of TGFβ, RANKL, and OPG, the three major cytokines that are involved in bone metastasis, at the TB- and TS-interfaces. The levels of TGFβ and RANKL were higher at the TB-interface compared with the TS-interface; hOCIF treatment did not suppress the levels of these cytokines (Figure 2E,F). Interestingly, hOCIF treatment significantly increased mOPG levels at the TB-interface, but it did not change mOPG levels at the TS-interface (Figure 2G). These results indicate that treatment with hOCIF significantly suppressed the degree of osteoclast induction, and osteolysis in the TB-microE, suggesting that increased mOPG may be involved in this effect.

### 2.3. Effects of hOCIF on the Induction of Necrosis and CSCs in the Microenvironments

**Figure 3:**
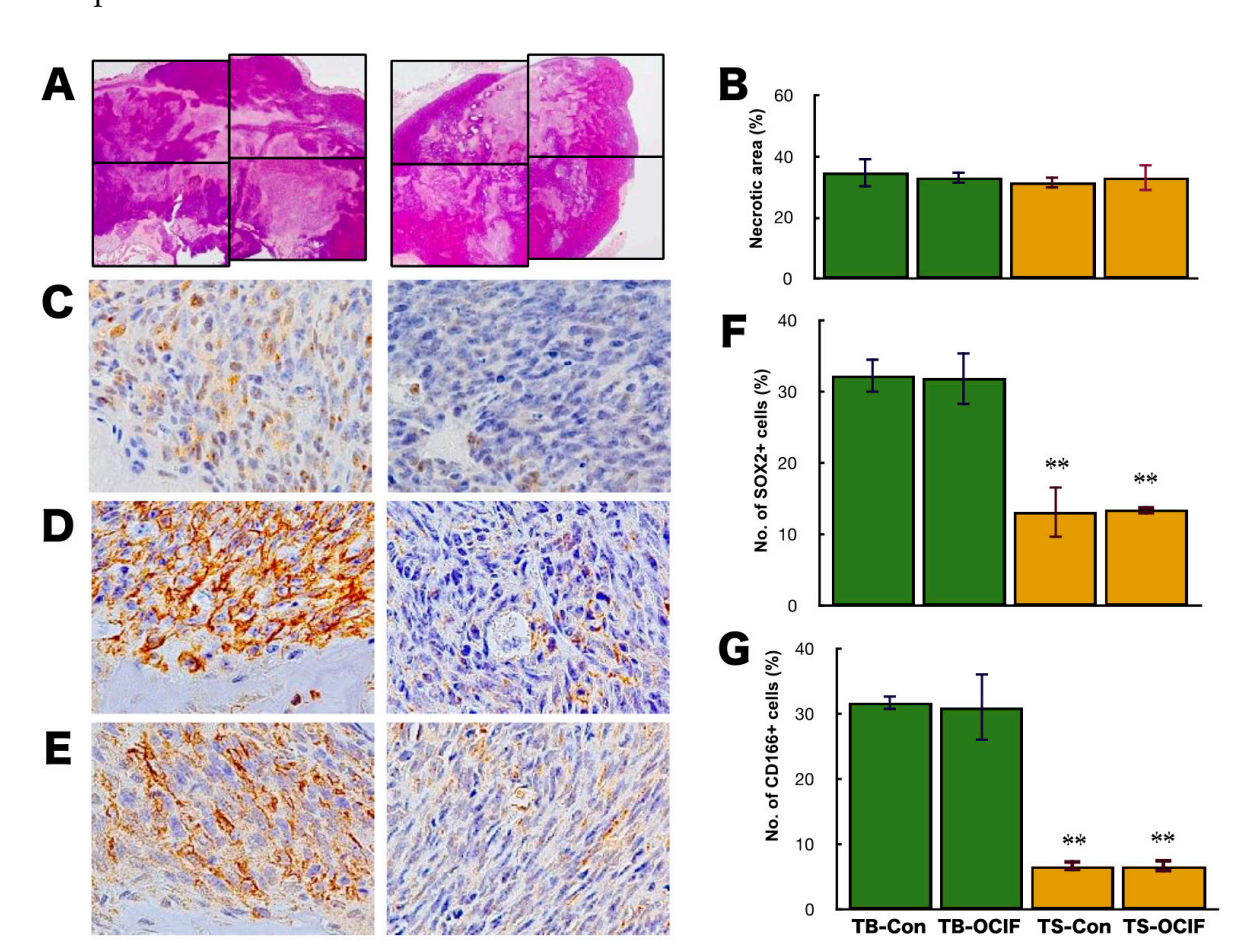
Effects of hOCIF on the induction of necrosis and CSCs in the tumor microenvironment. The necrotic area in the tumors was determined by microscopic analysis and image analyzer in the control and hOCIF groups (magnification ×1) (**A**). Quantitative analysis of the necrotic area revealed that hOCIF treatment did not affect the necrotic area (**B**). Among the available CSC markers, SOX2 (**C**), CD44 (**D**), and CD166 (**E**), positive cells were observed at both the TB- and TS-interfaces (magnification ×400). Quantitative analysis revealed that the number of SOX2 positive cells at the TB-interface was higher than those at the TS-interface. hOCIF treatment did not affect the number of SOX2 positive cells in either interface (**F**). Similar results were obtained for CD44 positive tumor cells (**G**). ** *p* < 0.01 vs. TB-Con.

Generally, the effectiveness of chemotherapeutic agents on cancer is evaluated by the increase in the necrotic area in the tumor tissues. Although the actual area of necrosis in the outgrowing tumor may increase, the ratio of necrotic area in the tumor (%) would not increase. If the tumor is sensitive to chemotherapeutic agents, the necrotic area (%) will increase, and if the tumor is resistant, the necrotic area (%) will not increase. To evaluate the effects of hOCIF on cranial and subcutaneous tumors, we examined the necrotic area in the tumors (%) by microscopic analysis and image analyzer (Figure 3A,B). Quantitative analysis of the necrotic area in the tumor revealed that hOCIF treatment did not affect the necrotic area (Figure 3B).

CSCs have been demonstrated to be involved in drug resistance, therefore we examined the effects of hOCIF on the induction of CSCs at the TB- and TS-interfaces. Among the available CSC markers, SOX2 [25] (Figure 3C), CD44 [26] (Figure 3D), and CD166 [27] (Figure 3E) positive cells were observed at both the TB- and TS-interfaces. Quantitative analysis of the number of SOX2 positive cells revealed that SOX2 positive cells at the TB-interface (Figure 3C left) was higher than at the TS-interface (Figure 3C right). hOCIF treatment did not affect the number of SOX2 positive cells at either interface (Figure 3F). Similar results were obtained for CD44 positive tumor cells (Figure 3G) and CD166 positive cells.

### 2.4. Preventive Effects of hOCIF on Tumor Growth and Cell Proliferation

**Figure 4:**
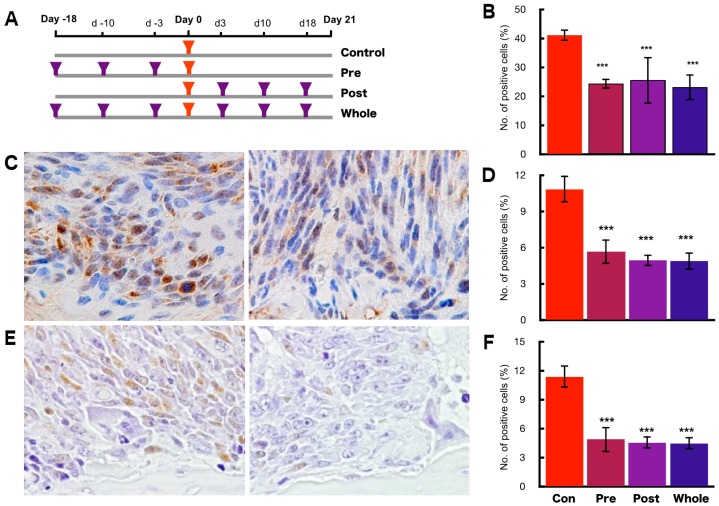
Preventive effects of hOCIF on bone-associated tumor growth in the bone microenvironment. (**A**) Mice were treated with hOCIF before tumor cell implantation (Pre group), after tumor cell implantation (Post group), and both before and after implantation (Whole group); (**B**) Quantitative analysis of PCNA positive cells at the TB- and TS-interfaces. Cell proliferation at the TB-interface evaluated by PCNA index was suppressed to the same extent in all three treatment groups; (**C**) Phospho ERK staining of the control group at the TB-interface (left, ×400) and hOCIF treatment group at the TB-interface (right, ×400); (**D**) Quantitative analysis of pERK positive tumor cells at the TB- and at the TS-interfaces. The index of pERK positive tumor cells was suppressed to the same extent in all three treatment groups; (**E**) Phospho NF-κB (pNFκB) positive tumor cells in the control at the TB-interface (left, ×400) and the hOCIF treatment group at the TB-interface (right, ×400); (**F**) Quantitative analysis of pNFκB positive tumor cells. The index of pNF-κB positive tumor cells was suppressed to the same extent in all three treatment groups. *** *p* < 0.001 vs. control.

To investigate the preventive effects of hOCIF at the TB-interface, mice were treated with hOCIF before tumor cell implantation (Pre group), after tumor cell implantation (Post group), and both before and after implantation (Whole group) (Figure 4A). As expected from the results shown in Figure 1B, hOCIF treatment suppressed tumor growth: Notably, tumor growth was suppressed to the same extent in all three treatment groups (Supplement Figure S2A). Next, we examined cell proliferation at the TB-interface (Figure 4B). Again, cell proliferation at the TB-interface was suppressed to the same extent in all three treatment groups (Figure 4B).

Next, we examined the association of signal transduction of Extracellular Signal-regulated Kinase (ERK) and nuclear factor-kappa B (NF-κB) with the suppressive effect of hOCIF on the cell proliferation at the TB-interface. We observed tumor cells strongly positive for phospho ERK (pERK) at the TB-interface in the control group (Figure 4C left) and hOCIF treatment significantly reduced the number of pERK positive cells at the TB-interface (Figure 4C right and D). Similarly, we observed many tumor cells positive for phospho IKKα (phosphor Inhibitor of nuclear factor kappa-B kinase subunit alpha, pIKKα) at the TB-interface in the control (Figure 4E left) and significantly fewer pIKKα positive cells at the TB-interface in the hOCIF treatment group. Thus, preventive effects on cell proliferation by hOCIF was observed in the TB-microE but not in the TS-microE. Suppression of cell proliferation by hOCIF was correlated with ERK and NF-κB signal transduction, suggesting that mechanisms of hOCIF-mediated suppression on tumor cell proliferation may involve these signaling pathways.

### 2.5. Preventive Effects of hOCIF on Osteolysis and Cytokine Levels in the Tumor Microenvironment

Quantitative analysis of osteolysis revealed that hOCIF treatment significantly suppressed the degree of osteolysis in the Pre, Post, and Whole groups (Supplement Figure S2C). We next evaluated the number of activated osteoclasts using TRAP staining (Supplement Figure S2D), and found that all three hOCIF treatments also suppressed osteoclast induction. Importantly, quantitative analysis of TRAP staining revealed that hOCIF significantly suppressed osteoclast induction in the TB-microE (Supplement Figure S2E).

**Figure 5:**
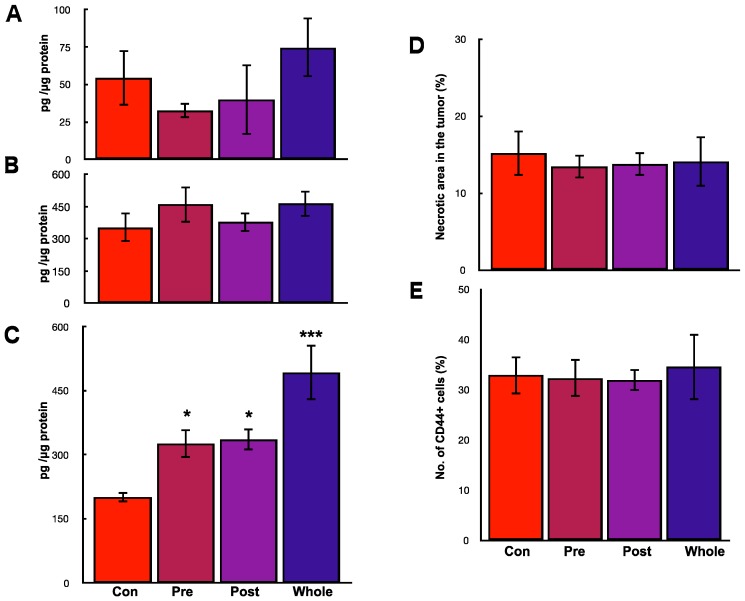
Preventive effects of hOCIF on cytokine levels, induction of necrosis, and CSCs in the microenvironments. hOCIF treatment did not affect the levels of TGFβ (**A**) or RANKL (**B**); (**C**) mOPG levels at the TB-interface were significantly increased in all three treatment groups; (**D**) The proportion of the necrotic area in the tumor was not changed in any of the three treatment groups. E: hOCIF did not affect the number of CD44 positive cells at the TB-interfaces. *, *** *p* < 0.05, *p* < 0.001 vs. control.

We measured the levels of TGFβ, RANKL, and mOPG at the TB-interface. hOCIF treatment did not affect the levels of TGFβ (Figure 5A) or RANKL (Figure 5B), however, mOPG levels at the TB-interface were significantly increased in all three treatment groups (Figure 5C).

### 2.6. Preventive Effects of hOCIF on the Induction of Necrosis and CSCs in the Tumor Microenvironment

Quantitative analysis of the necrotic area in the tumor revealed that the proportion of the necrotic area in the tumor was not changed in any of the three treatment groups (Figure 5D). Quantitative analysis of the number of CD44 positive cells revealed that hOCIF did not affect the number of CD44 positive cells (Figure 5E). Similar results were obtained with CD166 positive tumor cells.

All three treatments suppressed osteoclast activation, osteoloysis, and tumor growth and increased mOPG levels, but did not affect necrosis or induction of CSCs, suggesting that suppression of osteoclast activation, osteolysis, and tumor growth may be related to elevated mOPG levels in the TB-microE.

### 2.7. Preventive Effects of Low Doses of hOCIF on Osteolysis and Cytokine Levels in the Tumor Microenvironment (3)

**Figure 6:**
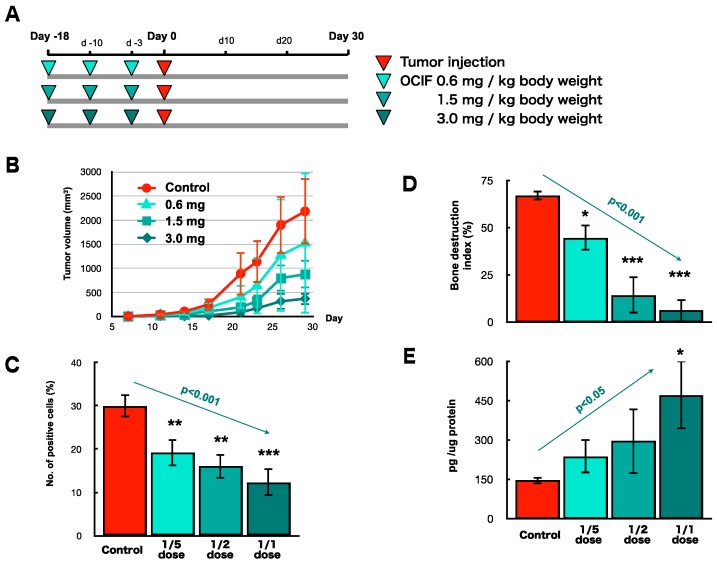
Preventive effects of low doses of hOCIF on tumor cell proliferation, osteolysis and cytokine levels in the tumor microenvironment. (**A**) We set 3.0 mg/kg body weight as the full dose, and examined the preventive effects of hOCIF at one-half and one-fifth dose of the full dose; (**B**) Pretreatment with hOCIF suppressed tumor volume in a dose dependent manner; (**C**) hOCIF treatment significantly suppressed cell proliferation at the TB-interface in a dose dependent manner (*p* < 0.001); (**D**) The bone destruction index was suppressed by pretreatment with hOCIF in a dose dependent manner; (**E**) mOPG levels at the TB-interface was increased in a dose dependent manner. *, **, *** *p* < 0.05, *p* < 0.01, *p* < 0.001.

Next, we examined the preventive effects of low doses of hOCIF. In the first and second experiments, we used 3.0 mg/kg body weight; therefore, in this series of experiments we pretreated mice with 0.6, 1.5, and 3.0 mg/kg body weight (Figure 6A). All three doses of hOCIF suppressed tumor volume (Figure 6B) and significantly suppressed cell proliferation at the TB-interface in a dose dependent manner (Figure 6C). The bone destruction index was also significantly suppressed by pretreatment of OCIF in a dose dependent manner (Figure 6D). In agreement with these results, osteoclasts induction was also suppressed by pretreatment with hOCIF, with the suppression being statistically significant in the 1.5 and 3.0 mg/kg groups (Figure 6E). The mOPG levels at the TB-interface were increased in a dose dependent manner and a significant increase was observed in the 3.0 mg/kg group (Figure 6F). Pretreatment with hOCIF did not suppressed TGFβ or RANKL levels at the TB-interface. These results suggest that pretreatment with hOCIF at low doses could suppress osteoclast induction and tumor growth in the TB-microE.

### 2.8. Effects of hOCIF and mOPG on Cell Proliferation of Mammary Tumor Cells In Vitro

**Figure 7:**
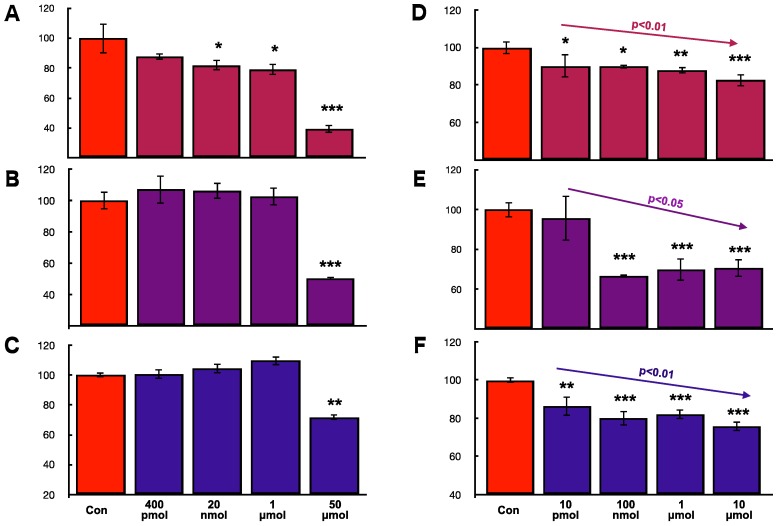
Effects of hOCIF and mOPG on cell proliferation of mammary tumor cells in vitro. To examine whether hOCIF is able to directly suppress cell proliferation, we examined the effect of hOCIF on the proliferation of 4T1 (**A**), CL66 (**B**), and CL66M2 (**C**) cells in vitro and the effect of mOPG on the proliferation of 4T1 (**D**), CL66 (**E**), and CL66M2 (**F**) cells in vitro. mOPG, but not hOCIF, suppressed the proliferation of all three cell lines in vitro, suggesting that mOPG but not hOCIF, may have directly suppressed the proliferation of mammary tumor cells in vivo. *, **, *** *p* < 0.05, *p* < 0.01, *p* < 0.001.

To examine whether hOCIF is able to directly suppress cell proliferation, we examined the effect of hOCIF on three different mouse mammary cell lines, 4T1 (Figure 7A), CL66 (Figure 7B), and CL66M2 (Figure 7C) in vitro. Although hOCIF treatment of the 20 nmol, 1 μmol, and 50 μmol groups significantly suppressed proliferation of 4T1 cells (Figure 7A), hOCIF did not suppress cell proliferation of CL66 (Figure 7B) or CL66M2 (Figure 7C) except in the 50 μmol/mL group probably due to toxicity. Next, we examined the effect of mOPG on 4T1 (Figure 7D), CL66 (Figure 7E), and CL66M2 (Figure 7F) cells. We found that proliferation of all three mammary cell lines, was significantly suppressed in a dose dependent manner by mOPG. These in vitro cell proliferation results suggest that, mOPG, but not hOCIF, may have directly suppressed the cell proliferation of mammary tumor cells in vivo.

### 2.9. Effects of hOCIF on Proliferation and mOPG Levels of Mammary Tumors Cell, Osteoclasts, and Mouse Mesenchymal Stem Cells In Vitro

**Figure 8:**
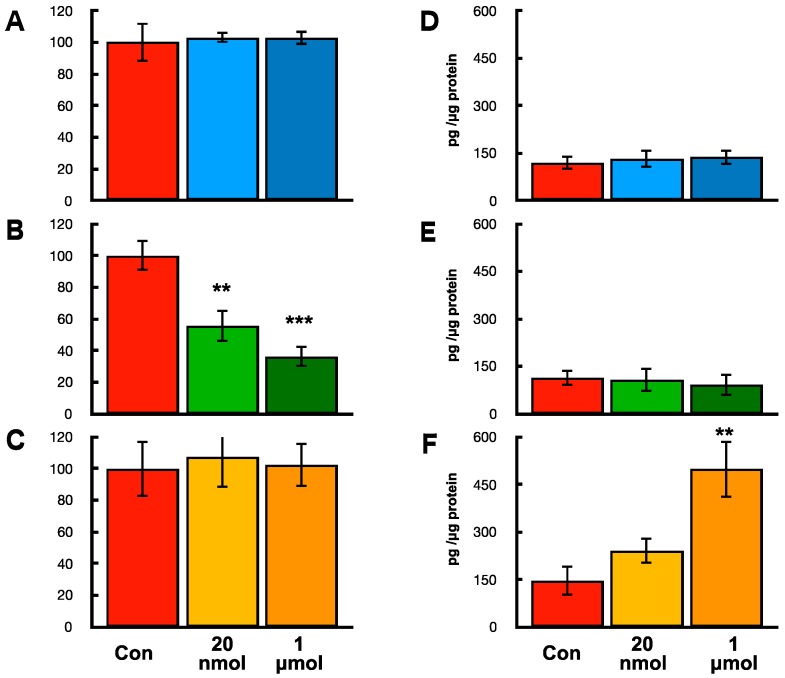
Effects of hOCIF on proliferation and mOPG levels in mammary tumors cells, osteoclasts and mouse mesenchymal stem cells in vitro. Effects of hOCIF on the proliferation of CL66M2 (**A**), RAW 264.3 cells (**B**), and hMSC cells (**C**). hOCIF suppressed the cell proliferation of RAW 264.3 cells in a dose dependent manner. Effects of hOCIF on mOPG level in CL66M2 (**D**), RAW 264.3 cells (**E**) and hMSC cells (**F**). A significant increase was observed in hMSC cells. **, *** *p* < 0.01, *p* < 0.001.

To determine which cells are responsible for the high levels of mOPG at the TB-interface in the hOCIF treatment group, mouse mammary tumor cell (CL66M2), osteoclasts (RAW 264.3), and mouse mesenchymal stem cells (mMSC) were incubated with hOCIF in vitro and cell proliferation and mOPG levels were examined (Figure 8). Treatment with 20 nmol and 1 μmol hOCIF did not suppress the proliferation of CL66M2 (Figure 8A) or hMSC cells (Figure 8C), however, hOCIF treatment suppressed the cell proliferation of RAW 264.3 cells in a dose dependent manner (Figure 8B). Treatment with hOCIF significantly increased mOPG levels in mMSC cells (Figure 8F), but not in CL66M2 (Figure 8D) or RAW 264.3 cells (Figure 8E). These results indicate that treatment of mouse mesenchymal stem cells with hOCIF in vitro resulted in the induction of mOPG expression, and suggests that this may be the mechanism by which hOCIF treatment increased mOPG levels in the TB microE in vivo.

## 3. Discussion

The lack of in vivo models that accurately simulate the progression commonly observed in patients with bone metastasis is a major obstacle to identifying the molecular mechanisms of bone metastasis and to developing therapies [28,29]. An ideal model of bone metastases in vivo should delineate the natural course and the progression observed in advanced cancer patients. The production of bone metastases in established models is achieved by left ventricular/intra-arterial injection of tumor cells [11,30], and mainly give rise to invasion, transport, arrest, adherence, and extravasation rather than growth in the microenvironment. It is now recognized that the bone microenvironment provides appropriate conditions for the survival and proliferation of the tumor cells [31,32]: the growth factors and cytokines produced in the bone microenvironment through tumor stromal interactions were previously demonstrated to promote the malignant behavior of tumor cells [33,34]. We have developed a rat bone invasion model of prostate cancer [9] and a mouse bone invasion model of breast cancer [24,35]. In our bone invasion models, tumor cells can be observed growing at the sites of osteolytic lesions mediated by activated osteoclasts at the TB-interface. Our “bone invasion model” does not delineate all the steps of bone metastasis, but focuses on the steps of survival and growth of the tumor in the bone microenvironment. Therefore, this model would elucidate the molecular mechanisms of survival and growth of the tumor in the bone microenvironment.

In the series of studies using our “bone invasion model”, we found that the invasion of the bone (bone destruction) was directly associated with osteoclasts, and it is widely accepted that acids produced by osteoclasts are responsible for degeneration of the bone matrix. In the present study, we found that treatment with hOCIF suppressed the induction of osteoclasts as well as bone destruction. Therefore, we believe treatment with hOCIF did not decrease the invasion of tumor cells.

It is possible that treatment with hOCIF may decrease the malignant potential of mammary tumor cells. Previously, we demonstrated TGFβ promoted the malignant potential of rat prostate cancer [36] and mouse mammary tumor [24] in the bone microenvironment. Since in the present study hOCIF did not decrease TGFβ levels in the TB-interface, treatment with hOCIF may not affect the invasiveness of the tumor cells; however, further studies are necessary to identify the factors other than TGFβ that may be involved in promoting the malignant potential of tumors.

Mouse TNFRSF11B (mOPG) shares 86%AA identity with human TNFRSF11B (hOCIF) [37], and human TNFRSF11B is known to be active in mice [38]. In the present study, we transplanted mouse mammary tumor cells onto the cranial bone and into a subcutaneous lesion in mice, and then treated the mice with hOCIF. We found that hOCIF suppressed tumor growth at the TB-interface but not at the TS-interface. hOCIF also suppressed osteolysis and the activation of osteoclasts at the TB-interface. Finally, hOCIF increased the levels of mOPG at the TB-interface. Taken together, these results suggest that hOCIF induced mOPG expression and that mOPG in turn inhibited activation of osteoclasts in the TB-microE.

OCIF/OPG is expressed in a wide variety of tissues and inhibits bone resorption in vivo as well as in vitro [37], and the mechanisms by which the production of OCIF/OPG is regulated in various cells are gradually becoming clarified. Production of OCIF/OPG is stimulated by TGFβ in bone marrow stromal cells [39], by prostate-specific antigen in human osteoblasts [40], and by phytoestrogen genistein in human trabecular osteoblasts [41], and is suppressed by basic fibroblast growth factor (bFGF) in human fibroblast-like synovial cells [42]. In the present study, because the level of TGFβ at the TB-interface was higher than that in TS-interface, it is possible that hOCIF treatment in association with TGFβ may be involved in the production of mOPG in the TB-microE. However, the totality of cell types that produce OCIF/OPG and the specifics of how its expression is regulated remains unclear; further detailed studies are required to elucidate the mechanism by which hOCIF increases the expression of mOPG.

Osteoclast differentiation was demonstrated to be modulated by RANKL-RANK signaling in cooperation with nuclear factor of activated T cells (NFAT) [43] and TGFβ [24,44,45]. RANK was also demonstrated to interact with the intracellular molecule TNF receptor–associated factor 6 (TRAF6), which plays an important role in the several signaling pathways, including NF-κB, p38 kinase, and c-Jun N-terminal kinase (JNK) [46]. Treatment with p38 inhibitors suppresses osteoclastogenesis in vitro [43], suggesting that p38 is involved in controlling osteoclast differentiation. Stimulation of JNK elicits the activation of the transcription factor c-Jun [47]. c-Jun forms activator protein-1 (AP-1) complexes with c-Fos, a transcription factor essential for osteoclast formation [48]: activation of c-Jun signaling was found to be essential for RANKL-regulated osteoclast formation both in vitro and in vivo [49]. In this study, we found that treatment with hOCIF which binds RANK as a decoy receptor significantly reduced the induction of osteoclasts in the TB-microE. By binding RANK, hOCIF prevents RANK-mediated activation of these pathways, thereby suppressing osteoclastogenesis. 

We also found that hOCIF suppressed the proliferation of mammary tumor cells at the TB-interface and that mOPG suppressed the proliferation of mammary tumor cells in vitro. Proliferation of lobulo-alveolar cells in the mammary grand during pregnancy is regulated by RANKL-RANK signaling [50] through activation of IκB kinase α (IKKα) [51]. IKKα has been previously demonstrated to be involved in the proliferation of prostate cancer cells, mammary cancer progenitors, and breast cancer cells [52,53,54].

Previous studies demonstrated important roles IKKα and extracellular signal-regulated kinase (ERK) phosphorylates as well as signaling pathways by receptor activator RANKL in osteoclast activation [55,56]. Our IHC study revealed that many tumor cells but very few osteoclasts are positive for phospho ERK and phospho IKKα, indicating activation of these signaling pathways. We also found that signal transduction of NF-κB and ERK in the tumor cells was down-regulated by the treatment with hOCIF, which was associated with suppressive effects on tumor cell proliferation by hOCIF. Therefore, our results suggest that RANKL-induced activation of ERK and NF-κB was observed in precursor of osteoclasts and tumor cells but not in mature osteoclasts. Because activation of ERK is involved in cell proliferation, down regulation of phospho ERK may be involved in the suppression of tumor cell proliferation by hOCIF.

Taken together, these results suggest that RANKL-RANK signaling may impact the proliferation of mammary tumor cells as well as osteoclast activation in the TB-microE. However, while hOCIF is known to be active in mice [38], the complete mechanism of action, including the totality of signaling pathways affected, how they are modulated, and which cells types are affected in the bone microenvironment when hOCIF or mOPG binds to RANK has not yet been completely elucidated.

The management of bone metastases can be accomplished by the combination of chemotherapy and radiation therapy plus bone modifying agents to prevent SREs [14]. However, progressive pain due to bone metastases can become unmanageable because abatement by analgesics, even opioid therapy, is frequently ineffective [16,17,57]. Thus, strategies to delay worsening of skeletal pain and aggravation of metastatic bone diseases are becoming increasingly important in the management of metastatic bone diseases. Programs have been initiated to prevent metastasis and cancer therapy-induced bone loss [58,59]. However, prolonged administration of these preventive agents can have serious side effects, such as necrosis of the jaw bone [60,61] during the preventive stage. We have demonstrated that hOCIF treatment at a low dose could be effective in preventing the development of bone destruction. Current guidelines recommend osteoporosis doses of BMA during adjuvant therapy and monthly dosing only to prevent SREs.

CSCs are regarded as being involved in the formation of metastases [62,63,64]. It is possible that CSCs in the bone microenvironment may explain drug resistance because of their slow growth rates [20]. If CSCs were a small minority in the tumors, treatment of the therapeutic agent would induce necrosis in non-CSCs which are a majority in tumors, and this treatment would increase the necrotic area in the tumor. If CSCs are not a minority in the tumors, treatment would not increase the necrotic area in the tumor. In the present study, we found that the bone microenvironment is a niche for CSCs. Therefore, to evaluate the response to therapy response by tumor, including CSCs, careful monitoring using apoptotic and proliferative markers is required because the correlation between the number of CSCs and induction of necrosis is complex.

We evaluated the response of the tumor following hOCIF treatment by the pathological size of the necrotic area of the tumor in this study. The necrotic area in the tumor was not affected by hOCIF in the present study, indicating that hOCIF treatment did not affect the number of CSCs at either the TB- or TS-interfaces. Further studies are necessary to investigate the tumor responses after chemotherapy targeting CSCs, which are coupled with inherent tumor heterogeneity [65].

In this study, we found that hOCIF treatment increased mOPG levels in mMSC cells in vitro. In the previous studies, serotonin significantly increased secretion of OPG and decreased that of RANKL secretion by bone marrow-derived mesenchymal stromal cells [66] and zoledronic acid (bisphosphonate) increased the proliferation and increased OPG expression by non-osteoblasts/mesenchymal cells in inhibition of osteoclast differentiation [67]. Because we found hOCIF treatment increased OPG levels in mMSC cells in vitro and in the TB-microE in vivo and significantly decreased the induction of osteoclasts, compounds that increase OPG levels in mesenchymal cell could decrease osteoclastgenesis. Taken altogether, these results suggest that treatment with hOCIF induced mouse mesenchymal cells to secrete mouse OPG in the bone microenvironment and secretion of mouse OPG in turn inhibited osteoclast differentiation.

In summary, treatment with hOCIF increased mOPG levels in the TB-microE, but not in the TS-microE, leading to suppressed activation of osteoclasts, suppression of osteolysis, and suppressed growth of mammary tumor in the bone microenvironment. mOPG suppressed mammary tumor cell proliferation in vitro, and likely suppressed mammary tumor cell proliferation in the bone microenvironment. hOCIF did not directly kill mammary tumor cells and did not affect CSCs in the bone microenvironment. Overall, RANKL is a potential target for the treatment and prevention of bone metastases of breast cancer.

## 4. Materials and Methods

### 4.1. Tumor Cell Lines and Tissue Preparation

4T1, Cl66 and Cl66M2 [28,68,69] were maintained in DMEM (Cellgro, Herndon, VA, USA) with 5% FBS and gentamycin at 37 °C in a humidified atmosphere containing 5% CO_2_. 1 × 10^5^ of Cl66M2 cells were transplanted mixed with growth factor-reduced Matrigel (BD Biosciences, San Jose, CA, USA) under the dorsal skin over the calvaria of female BALB/c mice. Mice were killed at 4 weeks post-transplantation for examination of osteolytic lesions. The tumor involving bone tissue were divided into two pieces. One piece was used for histology sections and the other piece was used for separation of the TB interface from the tumor-alone area for further analysis. Tissues were fixed with periodate-lysine-paraformaldehyde (PLP) at 4 °C for 48 h and then transferred into a decalcification solution (15% ethylene diamine tetra acetate with glycerol, pH 7.4–7.5) for 4 weeks, which was paraffin embedded and processed for histological analysis. All studies were done in accordance with the Institutional Animal Use and Care Committee of Graduate School of Medical Sciences, Nagoya City University (H22-M19, approved at 20 April 2010). 

### 4.2. Treatment with Human Recombinant OCIF (hOCIF)

To address the effects of hOCIF on tumor-induced osteolysis, Cl66M2 cells were transplanted onto the calvaria of BALB/c mice, and the mice were then treated with human recombinant OCIF (kindly provided by Daiichi Sankyo Co., Tokyo, Japan) at a dose of 2.0 mg/kg bodyweight six times during the experimental period. In the second set of experiments, mice were treated with hOCIF at a dose of 3.0 mg/kg body weight (b.w.) three times before transplantation (Pre group) or three times after transplantation (Post group) or three times before and three times after transplantaion (Whole). In the third set of experiments mice were treated with hOCIF at a dose of 3.0 mg/kg b.w. (full), 1.5 mg/kg b.w. and 0.6 mg/kg b.w. three times before transplantation.

Tumor growth was monitored twice per week. Animals were killed on day 21 and tissue processed for histochemical analysis.

### 4.3. Immunohistochemistry and Tartrate Resistant Acid Phosphatase Staining

For the in vivo detection of SOX2, CD44, CD166 and proliferative cell nuclear antigen (PCNA), tumor sections were evaluated by IHC of sections of the tumor. For the IHC studies, the following diluted primary antibodies were used: SOX2 (1:50, Cell Signal Technology Japan, Danvers, MA, USA), CD44 (1:500, Abcam Japan, Tokyo, Japan), CD166 (1:50, Cell Signal Technology Japan, Danvers, MA, USA), PCNA (1:200, sc-56, Santa Cruz, CA, USA). IHC staining was performed by an automatic IHC machine, Leica Bond-max (Leica Microsystems, Tokyo, Japan). Tartrate resistant acid phosphatase (TRAP) assays were carried out to detect activated osteoclasts in vivo (Sigma Chemicals, St. Louis, MO, USA).

IHC sections were examined under a light microscope for quantitative analysis. The numbers of positive cells for SOX2, CD44, CD166, and PCNA/nuclei were evaluated at a magnification of ×400 for each lesion. About 8000 nuclei per tumor were counted.

### 4.4. In Vitro Cell Proliferation

Cl66, Cl66M2, and 4T1 cells were seeded in 96-well plates at low density (1000 cells/well). Following overnight adherence, cells were incubated with different concentrations of recombinant mouse OPG or recombinant human OCIF for 72 h. 3-(4,5-Dimethylthiazol)-2,5-diphenylterazolium bromide (MTT) assay determined cell proliferation and growth was calculated as percent (%) = (1 − (A/B))× 100, where A and B were the absorbance of treated and untreated groups, respectively.

### 4.5. Effects of hOCIF on Osteoclast and Mouse Mesenchymal Stem Cell In Vitro

M2 cells, RAW 264.7 cells (ATCC TIB-71, Manassas, VA, USA) and mouse mesenchymal stem cells derived from the bone marrow of BALB/c mice (Cyagen, Santa Clara, CA, USA) were incubated in 6-well tissue culture plates. Adherent cells were washed extensively, then treated with minimum essential medium containing fetal bovine serum (FBS) alone (control) or containing hOCIF at 20 ng/mL or 1 mg/mL. Cells were treated every 3 days for 2 weeks and subsequently analyzed for cell proliferation and evaluation of OPG levels.

### 4.6. Statistical Analysis

The Kruskal-Wallis and Bonferroni-Dunn’s multiple comparison tests were used for in vivo, statistical analysis. The data are presented as means ± standard deviations. The statistical significance was analyzed using a two-tailed Student’s *t*-test and Bonferroni-Dunn’s multiple comparison tests and a value of *p* < 0.05 was considered significant. To determine dose-response correlation, the Spearman’s rank correlation test was used.

## Supplementary Materials

The following are available online at http://www.mdpi.com/1422-0067/19/3/888/s1.
