# Supplementary material for: Therapeutic and Preventive Effects of Osteoclastogenesis Inhibitory Factor on Osteolysis, Proliferation of Mammary Tumor Cell and Induction of Cancer Stem Cells in the Bone Microenvironment
Source: Int J Mol Sci. 2018 Mar 16;19(3):888. doi: 10.3390/ijms19030888 (PMC5877749; doi:10.3390/ijms19030888)
Supplement: Supplementary file 1 [file ijms-19-00888-s001.pdf]

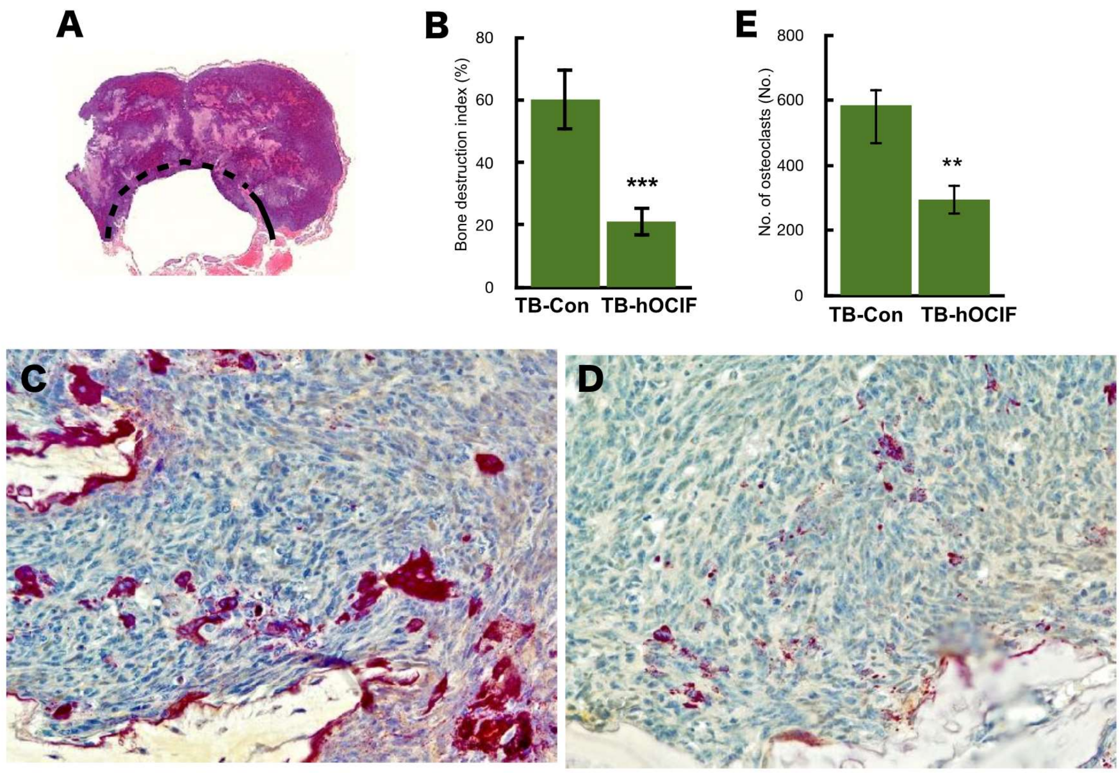

Supplement Figure S1. Effects of hOCIF on osteolysis in the tumor microenvironments A: Typical example of a defect in the cranial bone. The extent of bone destruction was determined using a 'bone destruction index', which is the ratio of the length of osteolysis to the length of the cranial bone. B: Quantitative analysis of the bone destruction index revealed that hOCIF significantly suppressed the degree of osteolysis. Numerous TRAP positive osteoclasts were observed in the control group (C), but only a few positive cells were observed in the hOCIF treatment group (D). Quantitative analysis revealed that hOCIF treatment significantly reduced the number of TRAP positive osteoclasts at the TB-interface (E). **, *** : $p < 0.01$, $p < 0.001$ vs. Control.

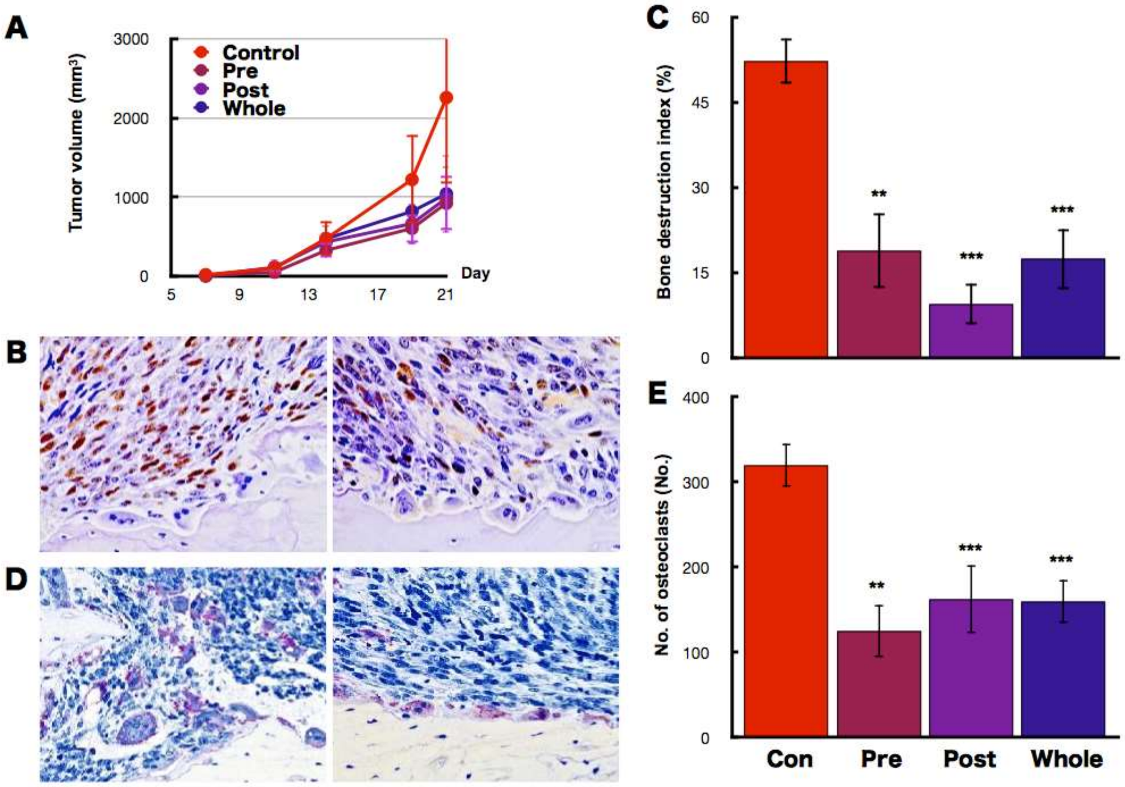

Supplement Figure S2: Preventive effects of hOCIF on bone-associated tumor growth, osteolysis, and activation of osteoclasts in the bone microenvironment. A: Tumor growth was suppressed to the same extent in the Pre, Post, and Whole groups.B: IHC study of PCNA in the control (left) and Pre groups (right). C: hOCIF treatment significantly suppressed the degree of osteolysis in the Pre, Post, and Whole groups. D: TRAP staining in the control (left) and Pre groups (right). E: hOCIF significantly suppressed osteoclast induction in the bone micro environment in all three treatment groups. **, *** : $p < 0.01$, $p < 0.001$ vs. Control.
